# CO_2_ hydrogenation to methanol over Pt functionalized Hf-UiO-67 versus Zr-UiO-67

**DOI:** 10.1098/rsta.2023.0269

**Published:** 2024-09-23

**Authors:** Dag Kristian Sannes, Sri Harsha Pulumati, Egill Skúlason, Ainara Nova, Unni Olsbye

**Affiliations:** ^1^ SMN Centre for Material Science and Nanotechnology, Department of Chemistry, University of Oslo, Oslo N-0315, Norway; ^2^ Science Institute and Faculty of Industrial Engineering, Mechanical Engineering and Computer Science, University of Iceland, Hjarðarhagi 2, VR-III, Reykjavík 107, Iceland; ^3^ Hylleraas Centre for Quantum Molecular Sciences, Department of Chemistry, University of Oslo, Oslo N-0315, Norway

**Keywords:** thermal catalysis, metal-organic frameworks, CO_2_ conversion, methanol, Hf-UiO-67, Zr-UiO-67

## Abstract

Sustainable methanol formation from CO_2_/H_2_ is potentially a key process in the post-fossil chemical industry. In this study, Hf- and Zr-based metal-organic framework (MOF) materials with UiO-67 topology, functionalized with Pt nanoparticles, have been tested for CO_2_ hydrogenation at 30 bar and 170–240°C. The highest methanol formation rate, 14 mol_methanol_ mol_Pt_^−1^ h^−1^, was obtained over a Hf-based catalyst, compared with the maximum of 6.2 mol_methanol_ mol_Pt_^−1^ h^−1^ for the best Zr-based analogue. However, changing the node metal did not significantly affect product distribution or apparent activation energy for methanol formation (44–52 kJ mol^−1^), strongly indicating that the higher activity of the Hf-based analogues is associated with a higher number of active sites. Both catalysts showed stable catalytic performance during testing under kinetic conditions, but the addition of 2 vol% water to the feed induced catalyst deactivation, in particular the Hf-MOFs. Interestingly, mainly methanol and methane formation rates decreased, while CO formation rates were less affected by deactivation. No direct correlation was found between catalytic stability and framework stability (crystallinity, specific surface area). Experimental and computational studies suggest that water adsorption strength to the MOF node may affect the relative catalytic stability of Hf-UiO-67-Pt versus Zr-UiO-67-Pt methanol catalysts.

This article is part of the discussion meeting issue ‘Green carbon for the chemical industry of the future’.

## 1. Introduction

Anthropogenic carbon dioxide (CO_2_) is a major contributor to the increase in atmospheric temperature and ocean acidification [1], and therefore, numerous research groups worldwide are now focusing on carbon capture and sequestration (CCS) or carbon capture and utilization (CCU) [2]. CCU has an additional benefit, besides just reducing the CO_2_ concentration in the atmosphere, of providing a renewable carbon source of fuels and high-value-added chemicals, which may lead to closing the carbon cycle [3]. CO_2_ utilization is challenging from a thermodynamic perspective due to the high stability of CO_2_, and often, a co-reactant with high energy, such as H_2_, is required to overcome the thermodynamic restraints [4].

The most investigated catalysts for CO_2_ hydrogenation are supported metal catalysts [5–7]. Among them, copper supported on ZrO_2_ is among the most frequently studied material [8–10]. Density functional theory (DFT) calculations have shown that the active site for the formation of methanol is at the interface of Cu nanoparticles (NPs) and ZrO_2_. Cu NPs can dissociatively adsorb H_2_, which facilitates the spillover and hydrogenation of CO_2_, adsorbed on ZrO_2_ in the proximity of the NPs. The most favourable pathway for methanol formation is through formate, as experimentally supported by isotopic labelling experiments [9].

In recent studies, metal-organic frameworks (MOFs) have received increased attention as potential support materials for CO_2_ hydrogenation catalysts due to their immense tunability, high surface areas and the possibility to finely tune the active sites in these highly crystalline materials [11]. Amidst the most studied MOFs are the UiO-type MOFs [12], especially, UiO-66 and UiO-67 due to their high stability [13]. The secondary building unit of the UiO-type MOFs is the zirconium oxocluster, Zr_6_O_4_(OH)_4_^12+^. The cluster consists of six Zr^4+^ in an octahedral arrangement, with each facet capped by either μ_3_–O or μ_3_–OH groups in an alternating fashion. The linkers of UiO-66 and UiO-67 are 1,4-benzenedicarboxylic acid and 4,4′-biphenyldicarboxylic acid (BPDC), respectively [14].

The synthesis and reactivity of UiO-67 functionalized with Pt NPs have been previously reported by our group [15–18]. In those investigations, the UiO-67-Pt material exhibited remarkable stability and selectivity to methanol, surpassing reference materials Pt/Al_2_O_3_, Pt/SiO_2_ and Pt/C (carbon black) [15]. However, the activity of the catalyst was limited due to poisoning by CO, which was observed using *in situ* infrared (IR) spectroscopy and kinetic studies. The active sites for methanol formation were found to be at the interface of Pt NPs and the zirconium nodes. Bidentate formate species observed using in operando IR spectroscopy during transient H/D experiments suggest that methanol is formed through a formate intermediate pathway. The importance of the open zirconium sites was evaluated by utilizing post-synthetic linker insertion on a Zr-UiO-67-Pt sample to reduce the number of open zirconium sites. The quantity of non-structural ligands was reduced by 60%, resulting in a 60% decrease in methanol and methane formation rates. The rate of CO formation was only affected to a small degree, indicating that open zirconium sites are not involved in the rate-determining step (RDS) of CO formation [17]. To investigate the mechanism of formation for methanol, methane and CO, a thorough DFT investigation was carried out [18]. Herein, it was found that the RDS of methanol formation is the hydride transfer to a formate species, which is further supported experimentally by the observation of an inverse isotope effect. The study found that methanol is formed at the interface of a zirconium cluster and an edge on the Pt NPs. Methane was found to be a secondary product of CO, first formed at the interface of a zirconium cluster and a Pt(111) surface, which could subsequently migrate to the Pt(111) surface for further hydrogenation to methane. The postulated mechanisms were supported by experimental results, where pyrolysis was used to tune the size of the Pt NPs. The material with large Pt NPs, which contains a large fraction of the flat Pt(111) surface, was more active for methane formation. By contrast, the material with small NPs was more active for methanol production as the material has a larger fraction of Pt edges. In summary, the experimental findings, complemented by DFT calculations, advanced the understanding of the mechanism of CO_2_ hydrogenation over Pt-functionalized UiO-67.

Since the discovery of UiO-66 and UiO-67 in 2008, many research endeavours have been directed at unravelling isoreticular materials with similar stability but with new and interesting properties, e.g. replacing zirconium with similar elements [19–21]. Hafnium was an obvious choice, being below zirconium in the periodic table and the first hafnium equivalent of UiO-66 was reported in 2012 [22]. Due to the lanthanoid contraction, hafnium and zirconium are very similar in size. However, Hf is more oxophilic than Zr, with a dissociation enthalpy of 802 kJ mol^−1^ for the Hf–O bond and 776 kJ mol^−1^ for the Zr–O bond [23]. The increased bond strength between the node and the linkers suggests that hafnium MOFs should be more thermally stable than their Zr equivalents. However, studies on the thermal stability of Hf- and Zr-UiO-66 suggest that their thermal degradation temperature is virtually the same [24]. For a lower connectivity MOF, such as MOF-808, where each node is coordinated to eight linkers, the hafnium variant demonstrated an increase in thermal and mechanical stability over the zirconium variant, suggesting that for certain MOFs, the use of hafnium may provide more stable frameworks [24]. A universal method for determining materials’ chemical stability does not exist. Hence, comparing stability between different studies is difficult. Due to the stronger metal–O bond of Hf, it is postulated that Hf-MOFs could be more stable towards water than their Zr variant [25].

The increased oxophilicity of hafnium compared with zirconium is interesting from a catalytic perspective due to the increased Brønsted acidity of the hafnium node. Kobayashi *et al*. showed that the rate of CO_2_ hydrogenation over copper-functionalized Hf-UiO-66 is three times higher per gram of copper than copper-functionalized Zr-UiO-66 at 2 atm, 220°C and 5/1 ratio of H_2_ to CO_2_. They postulated that this increase in formation rate is due to the charge transfer capability of the hafnium node, facilitating the formation of methanol [26]. Similarly, when Rojas-Buzo *et al*. investigated Hf- and Zr-UiO-66 and MOF-808 for cross-aldol condensation with acetone, they observed a higher turnover frequency (TOF) per mol of node metal for the hafnium variants. Based on mechanistic studies using nuclear magnetic resonance (NMR) and isotopically labelled acetone, it was postulated that the increased activity of the hafnium samples was caused by their higher Lewis acid strength [27].

In this study, hafnium and zirconium UiO-67 materials were synthesized, functionalized with platinum and tested for CO_2_ hydrogenation to investigate the influence of the node metal on CO_2_ conversion and product distribution. Furthermore, the catalytic stability of the catalysts in the presence of water in the reaction feed was investigated. The synthesized materials were characterized using powder X-ray diffraction (PXRD), microwave plasma atomic emission spectroscopy (MP-AES), thermogravimetric analysis (TGA), ^1^H nuclear magnetic resonance (^1^H NMR), nitrogen adsorption and scanning electron microscopy (SEM). Furthermore, DFT calculations were performed to investigate water adsorption strength on hafnium nodes, as done in a previous work of the group for zirconium nodes [17]. The results of these studies suggest that, overall, replacing Zr with Hf does not provide a clear improvement in the catalytic properties of UiO-67-Pt for the CO_2_-to-methanol reaction.

## 2. Experimental details

Four samples were investigated in this study. Among them, three samples were synthesized in-house and one sample was purchased from PROFMOF A/S. The general synthesis procedure is given below, and the amount of each reactant for each synthesis is given in the electronic supplementary material, table S1. For all samples, 10% of BPDC was substituted with 2,2′-bipyridine-5,5′-dicarboxylic acid (BPYDC). These materials are denoted as Hf-UiO-67-FA, Hf-UiO-67-BA, Zr-UiO-67-FA and Zr-UiO-67-BA, where FA and BA indicate the use of formic acid and benzoic acid modulator, respectively, in the synthesis mixture. The procedure of incorporating Pt on the BPYDC sites was the same for all four samples, and the procedure was adapted from a previous publication [15]. The samples are denoted by adding a –Pt to their names, e.g. Hf-UiO-67-FA-Pt. Linker insertion was performed on both UiO-67-FA-Pt samples to evaluate the effect of ligand exchange. The procedure for linker insertion was the same for both materials.

DMF and the modulator (either formic acid or benzoic acid) were added to a round bottom flask and followed by either ZrCl_4_ or HfCl_4_ addition. The BPDC was then dissolved in the solution, and the mixtures were heated at 120°C for 24 h before filtration. The isolated material was dispersed in 20 ml hot DMF and carefully stirred for 5 min before being filtrated again. This was performed a total of two times with DMF and three times with acetone. The products were dried at 120°C for 24 h.

The procedure of incorporation of Pt onto the BPYDC sites was the same for the four materials, where 1 molar equivalent of the parent MOF (Zr_6_O_4_(OH)_4_(BPDC)_5.4_(BIPY)_0.6_, corresponding to 0.6 molar equivalents of BPYDC (refer to §4, vide infra), was dispersed in roughly 800 molar equivalents of DMF. The 0.3 molar equivalents of K_2_PtCl_4_ were added (equivalent to half the BPYDC sites), and the solution was heated under reflux at 100°C for 24 h. The isolated material was dispersed in 20 ml hot DMF and carefully stirred for 5 min before being filtrated again. This was performed a total of two times with DMF and three times with acetone. The products were dried at 120°C for 24 h. The metalation procedure changed the colour of the sample from white to bright yellow.

Linker insertion was performed to replace the monodentate capping agents with BPDC to reduce the fraction of missing linker defects in the formate materials. One molar equivalent of MOF and 1 molar equivalent of BPDC were dispersed in approximately 800 molar equivalents of DMF and heated at 100°C overnight. The excess BPDC corresponds to 17% of the BPDC of an ideal UiO-67 sample. The isolated material was dispersed in 20 ml hot DMF and carefully stirred for 5 min before being filtrated again. This was performed a total of two times with DMF and three times with acetone. The products were dried at 120°C for 24 h. The materials are denoted as *x*–UiO-67-FA-Pt (LD), where *x* is either Zr or Hf. The mentioned above procedure, except for adding BPDC to the reaction mixture, was performed on Hf-UiO-67-BA-Pt to prepare a blank sample, denoted Hf-UiO-67-BA-Pt-blank, to evaluate the effect of the procedure itself.

The structure and crystallinity of the synthesized materials were determined with PXRD using a Bruker D8 Discover instrument equipped with a Lynxeye detector and Ge(111) Johansson monochromator to selectivity use Cu_Kα1_ radiation. Experiments were performed from 2θ = 2–50 with step size and speed of 0.019° and 1 s, respectively. The thermal stability of the samples was determined using TGA conducted on a Netzsh STA 449 F3-Jupiter instrument. Experiments were performed in an alumina crucible from 30°C to 800°C using a ramp of 10°C min^−1^ and a flow of 100 ml min^−1^ of synthetic air. The porosity of the samples was determined using nitrogen adsorption isotherms obtained at −196°C using a BelSorp mini X instrument after pretreatment of the samples at 200°C for 2 h. Brunauer Emmet Teller (BET) theory was used to determine the specific surface areas for the materials. ^1^H NMR spectra of the digested materials were obtained to determine the quantity of BPYDC and combined with TGA to determine the number of missing linker defects [28]. The spectra were collected using a Bruker AVIII HD 400 instrument equipped with an autosampler. The digestion method is described in the electronic supplementary material [28]. MP-AES experiments were conducted using an Agilent 4100 MP-AES spectrometer, and the digestion procedure is described in the electronic supplementary material. SEM was used to determine the particles’ morphology and evaluate the potential Pt NPs sintering. SEM images were obtained using a Hitachi SU8230 Field Emission Scanning Electron Microscope using a voltage of 1 to 2 kV and an electron current of 10 µA.

Catalytic testing was performed using a silicon-coated, stainless-steel fixed-bed reactor with an internal diameter of 6 mm inside a Microactivity Effi (MAE) reactor built by PID Eng & Tech. The reactor was connected to a gas chromatograph equipped with two flame ionization detectors (FIDs) and one thermal conductivity detector. A methanizer was installed prior to one of the FIDs to lower the detection limits of CO and CO_2_. An Azura P 4.1S high-pressure liquid chromatograph pump from Knauer was used to feed 0.001 ml min^−1^ of liquid water into the MAE reactor, corresponding to 1.3 ml min^−1^ of water vapour when evaporated. For all experiments performed in this study, 0.2 g of the catalysts were reduced *in situ* at 350°C in 10% H_2_ in inert for 4 h using a temperature ramp of 10°C min^−1^. The catalytic testing was performed for 4 h for each test condition investigated, and all catalytic results, except TOS plots, are given as averaged values. All experiments presented herein were performed at 30 bars using a ratio of 1/6/2 of CO_2_/H_2_/Ar. To determine the formation rates of CO, methane and methanol, a contact time (τ) of 0.011 g_cat_ min ml^−1^ and a total flow of 18 ml min^−1^ were used. The apparent activation energy for methanol formation was determined by varying the contact time between 0.0056 and 0.0033 g_cat_ min ml^−1^ (1100–740 g_cat_ min mol_CO2_^−1^), and estimating the formation rate at contact time = 0 by extrapolation at 170–240°C. The values obtained were introduced into Arrhenius plots. The stability of the samples during these tests was verified by returning to the initial temperature after temperature variation at each contact time. The activity and stability of the synthesized materials, using 2 vol% of water in the reaction feed, have been investigated. To evaluate the effect of water on both the material’s catalytic properties and the materials’ stability, the materials were first tested without water, then with water co-feed and finally without water at 30 bars and in the temperature range of 170–240°C using a ratio of 1/6/2 of CO_2_/H_2_/Ar (*τ* = 0.0033 g_cat_ min ml^−1^). For comparison, water-free experiments were performed under the same conditions.

## 3. Computational details

All DFT calculations in this study were performed using the CP2K—8.1 package, with the Quickstep module [29] for energy and force evaluations, using the Gaussian and plane wave method [30,31]. Energies are calculated through the standard diagonalization algorithm and ELPA library [32]. PBE functional [33] with the DZVP-MOLOPT-SR-GTH basis set and Goedecker, Teter and Hutter (GTH) pseudopotentials [34] were used to represent core electrons, while double zeta basis functions represent valence electrons. A multi-grid of size 5 was used to map the Gaussian basis functions, with a cutoff energy of 360 Ry for plane wave basis and a grid level progression factor of 3. A relative cutoff of 60 Ry was used to determine which grid the Gaussian function should be mapped to. Dispersion corrections are implemented using Grimme’s DFT-D3 approach [35,36]. The Brillion zone was sampled at the Gamma point, and a convergence cutoff of 10^−6^ was used for all geometry optimizations and vibrational analysis.

BFGS optimizer was used for optimizing atomic coordinates and convergence criteria of maximum and RMS displacement, and maximum and RMS forces are below 3.0 × 10^−3^, 1.5 × 10^−3^ Bohr and 4.5 × 10^−4^, 3.0 × 10^−4^ Bohr^−1^ Hartree, respectively. Free energies were calculated for all gas phase species and adsorbed species on the active sites. In the case of adsorbed species, only the vibrational contribution was considered for the atoms in the adsorbates by computing a numerical partial Hessian. A central difference method with a displacement criterion of ±0.015 Å was computed in *x*, *y* and *z* coordinates. Thermal corrections for Gibbs free energies were computed from frequencies obtained from vibrational analysis using the Shermo package [37], where translational and rotational contributions were ignored for the adsorbed species, and only vibrational contribution was included. Micro kinetic models were built with the COPASI 4.27 software, and the time-course simulations were performed using the LSODA algorithm [38,39]. We used energy barriers of 0.5 kJ mol^−1^ for the adsorption processes with 0 kJ mol^−1^ barriers in the microkinetic models (MKMs).

## 4. Results

### (a) Characterization

PXRD was performed on all four parent materials synthesized in this work, and the results are plotted in the electronic supplementary material, figure S1. A Rietveld analysis against the known CIF file of UiO-67 was performed to determine the identity of the synthesized samples. The observed congruence provides strong evidence that the samples are indeed UiO-67. TGA and ^1^H NMR were performed to determine the composition of the materials using a previously published procedure [28], and the results are shown in the electronic supplementary material, figure S2 and table S2, respectively. The TGA results showed that both Hf- and Zr-UiO-67 decomposed at approximately 500°C in synthetic air. As expected, the molar mass of the benzoate MOFs is larger than that of the formate equivalents due to the much larger molar mass of benzoate compared with formate. The NMR results showed that approximately 10% of BPYDC was incorporated for all materials. The calculated chemical formulas for the synthesized materials are shown in table 1, and the hafnium samples contain fewer missing linker defects than their zirconium equivalents. The porosity of the samples was measured using nitrogen adsorption measurements; the results are shown in the electronic supplementary material, figure S3. The uptake of N_2_ of Zr-UiO-67-FA is lower than expected for the pristine material, while Zr-UiO-67-BA shows similar uptake to theoretical values. As expected, due to the increased molar mass of hafnium, both Hf-UiO-67 samples show reduced specific N_2_ uptake compared with the Zr-UiO-67-BA sample.

**Table 1.** Calculated chemical formulas for the synthesized materials based on TGA results in the electronic supplementary material, figure S2. ^1^H NMR results are shown in the electronic supplementary material, table S2.

sample	species per node	linker defects
Hf-UiO-67-FA	BPDC_4.87_BIPY_0.63_FA_0.19_ H_2_O/OH^–^_0.80_	1.0
Hf-UiO-67-BA	BPDC_4.82_BIPY_0.49_BA_0.68_FA_0.05_ H_2_O/OH^–^_0.65_	1.4
Zr-UiO-67-FA	BPDC_4.58_BIPY_0.50_FA_0.32_ H_2_O/OH^–^_1.52_	1.8
Zr-UiO-67-BA	BPDC_4.55_BIPY_0.45_BA_0.55_FA_0.09_ H_2_O/OH^–^_1.36_	2.0

PXRD was performed on the metalated samples to ensure that the post-synthetic metalation had not largely affected the structure. The results are presented in the electronic supplementary material, figure S4, and the diffractograms are still those of UiO-67 and similar to the parent materials’ diffractograms. Nitrogen adsorption isotherms were obtained on the metallated samples to investigate whether the porosity was affected. The results are shown in the electronic supplementary material, figure S5; only the Zr-UiO-67-BA-Pt sample showed a decrease in N_2_ uptake of approximately 15%. The Pt content for each material was determined using MP-AES and is reported in the electronic supplementary material, table S3.

To determine the effect of linker insertion, ^1^H NMR and TGA before and after linker insertion are shown in the electronic supplementary material, table S4 and figures S6 and S7, respectively. The larger the relative mass of the linker-inserted materials indicates that the heavier BPDC linker has replaced the formate capping species and the H_2_O/OH^–^ pairs. This is further supported by the NMR results, which show that the relative amount of formate has been reduced, and materials are suited to investigate the effects of reduced missing linker defects.

### (b) Catalytic investigation

**Figure 1.**
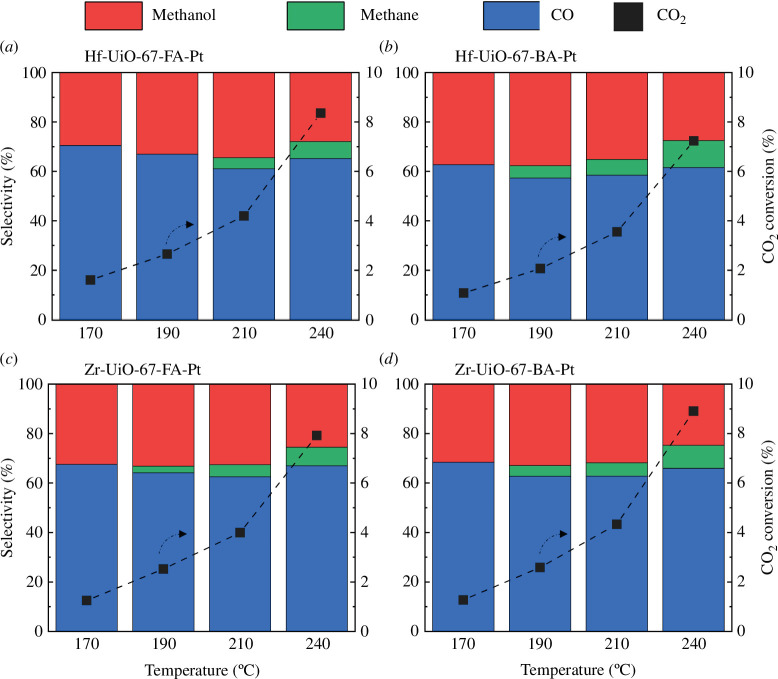
Evolution of selectivity to the products with temperature (columns, left axis) and the corresponding CO_2_ conversion (squares, right axis). (*a*) Hf-UiO-67-FA-Pt, (*b*) Hf-UiO-67-BA-Pt, (*c*) Zr-UiO-67-FA-Pt and (*d*) Zr-UiO-67-BA-Pt at 30 bars and a ratio of 1/6/2 of CO_2_/H_2_/Ar (*τ* = 0.011 g_cat_ min ml^−1^). The time on stream (TOS) for each test temperature tested was 4 h.

**Figure 2.**
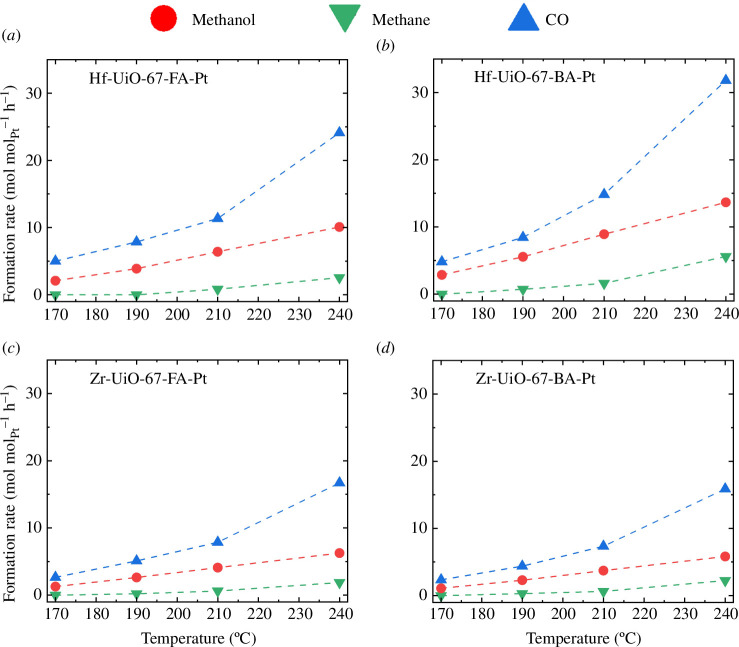
Formation rate (mol mol_pt_^−1^ h^−1^) of the products for (*a*) Hf-UiO-67-FA-Pt, (*b*) Hf-UiO-67-BA-Pt, (*c*) Zr-UiO-67-FA-Pt and (*d*) Zr-UiO-67-BA-Pt at 30 bars and a ratio of 1/6/2 of CO_2_/H_2_/Ar (*τ* = 0.011 g_cat_ min ml^–1^). The TOS for each test temperature tested was 4 h.

Conversion and selectivity plots as a function of temperature are shown in figure 1, and the formation rate of the products, reported in mol_product_ mol_Pt_^−1^ h^−1^, are shown in figure 2. Notably, CO_2_ conversion versus temperature and product distribution were similar for all samples. Likewise, the apparent activation energy for methanol formation was similar over the four materials (table 2 and in the electronic supplementary material, figure S8). Referring the methanol formation rate to the amount of H_2_-activating metal, Pt, similarly to Kobayashi *et al*. [26], a higher rate was observed for the Hf-based materials (figure 2). No significant loss of crystallinity is observed for the materials after catalytic testing at 30 bars and 170–240°C (electronic supplementary material, figure S9).

**Table 2.** Experimentally determined apparent activation energy (kJ mol^−1^) for methanol formation over Zr-UiO-67-BA-Pt, Zr-UiO-67-FA-Pt, Hf-UiO-67-BA-Pt and Hf-UiO-67-FA-Pt.

sample	apparent activation energy (kJ mol^−1^)
Zr-UiO-67-BA-Pt	51
Zr-UiO-67-FA-Pt	51
Hf-UiO-67-BA-Pt	44
Hf-UiO-67-FA-Pt	52

### (c) Catalytic stability

**Figure 3.**
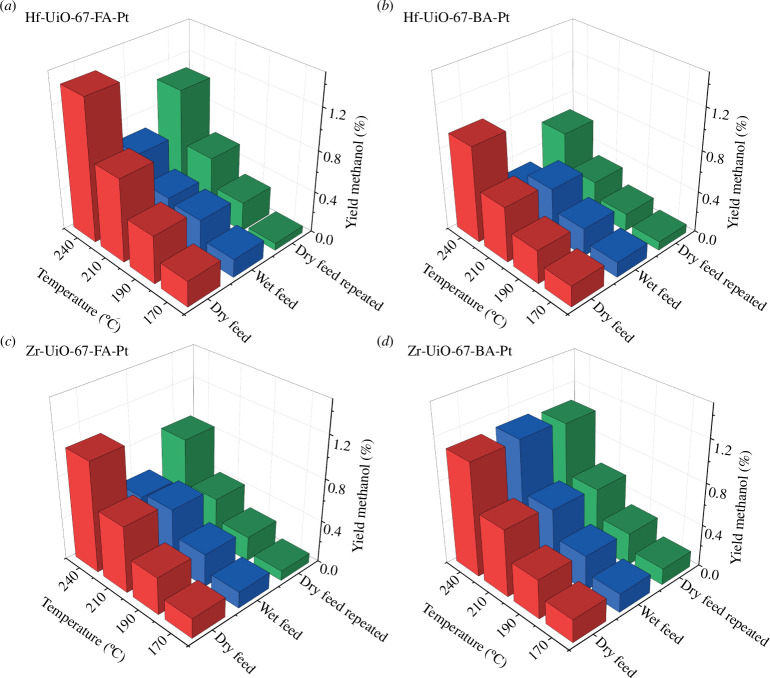
Evolution with temperature of methanol yield for the dry feed, wet feed and dry feed repeated for (*a*) Hf-UiO-67-Fa-Pt, (*b*) Hf-UiO-67-BA-Pt, (*c*) Zr-UiO-67-FA-Pt and (*d*) Zr-UiO-67-BA-Pt at 30 bars and a ratio of 1/6/2 of CO_2_/H_2_/Ar (*τ* = 0.0033 g_cat_ min ml^−1^). For the wet feed, water vapour constituted 2% of the total flow. The TOS for each test temperature tested was 4 h.

**Figure 4.**
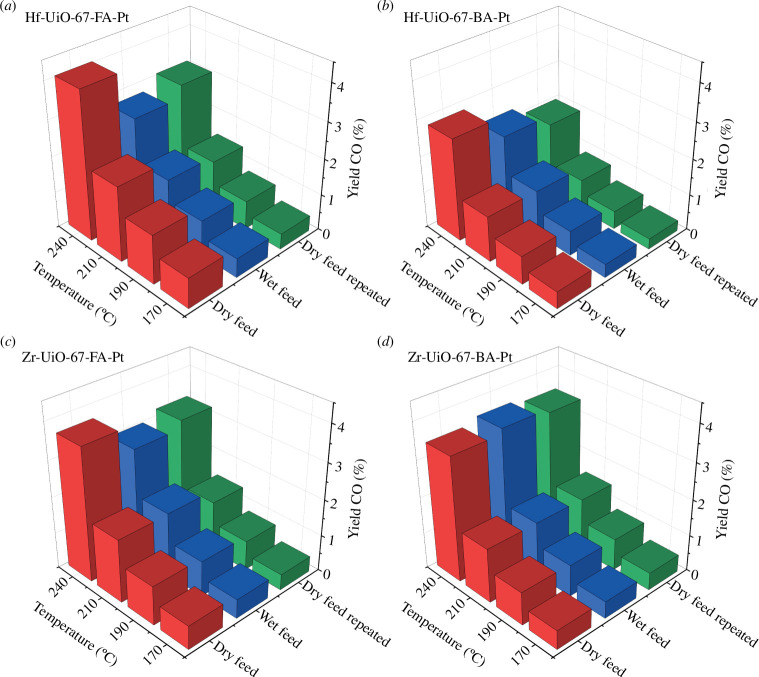
Evolution with temperature of CO yield for the dry feed, wet feed and dry feed repeated for (*a*) Hf-UiO-67-Fa-Pt, (*b*) Hf-UiO-67-BA-Pt, (*c*) Zr-UiO-67-FA-Pt and (*d*) Zr-UiO-67-BA-Pt at 30 bars and a ratio of 1/6/2 of CO_2_/H_2_/Ar (*τ* = 0.0033 g_cat_ min ml^−1^). For the wet feed, water vapour constituted 2% of the total flow. The TOS for each test temperature tested was 4 h.

Plots representing methanol yields versus temperature over the four samples during testing at different conditions (dry feed, subsequent wet feed and final dry feed) are shown in figure 3. The corresponding plots for CO and methane are shown in figure 4 and in the electronic supplementary material, figure S10, respectively. In addition, for each sample, the TOS performance during the water co-feeding experiment is represented in the electronic supplementary material, figures S11–S15, and the conversion and selectivity plots for all experiments are shown in the electronic supplementary material, figures S16–S19.

For all materials except Zr-UiO-67-BA-Pt, a decay in the yield of methanol and CO is observed during and after water is co-fed (figures 3 and 4, electronic supplementary material, figure S11). For Hf-UiO-67-FA-Pt, both before and after feeding water, the yield of methanol increases as the temperature increases. However, in the wet feed, a sudden decrease in the yield of methanol is observed after 10 h (i.e. when the sample has reached 210°C). Similarly, for Hf-UiO-67-BA-Pt and Zr-UiO-67-FA-Pt, the same loss of methanol yield is observed after 12 h on stream (240°C). No sudden loss of CO yield is observed over any materials during testing in the wet feed.

**Figure 5.**
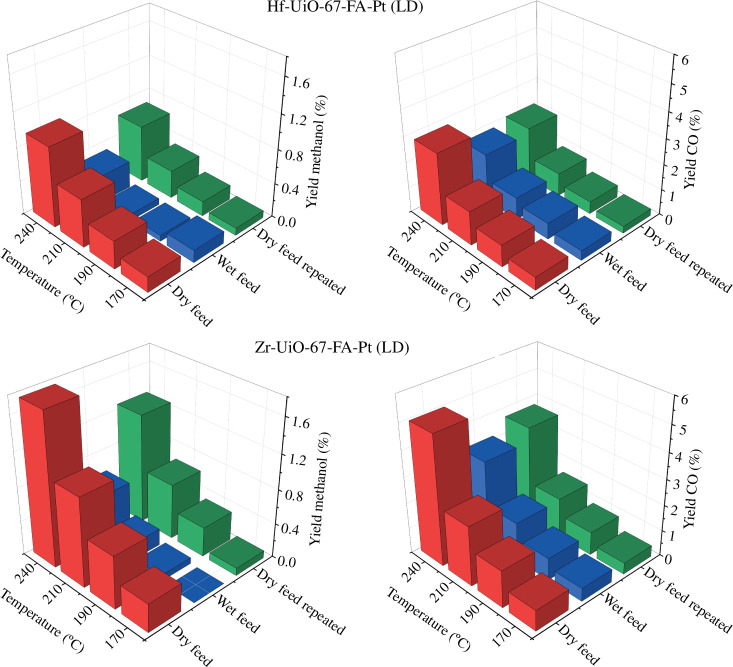
Evolution with temperature of methanol yield (left figures) and CO yield (right figures) for the dry feed, wet feed and dry feed repeated for Hf-UiO-67-Fa-Pt (LD) (top figures) and Zr-UiO-67-FA-Pt (LD) (bottom figures) at 30 bars and a ratio of 1/6/2 of CO_2_/H_2_/Ar (*τ* = 0.0033 g_cat_ min ml^−1^). For the wet feed, water vapour constituted 2% of the total flow. The TOS for each test temperature tested was 4 h.

Considering next the catalytic performance after wet feeding, i.e. under repeated dry feed conditions, electronic supplementary material, figure S12 shows the TOS performance, akin to electronic supplementary material, figure S11, for Hf-UiO-67-FA-Pt before and after water is co-feed. The catalytic testing results of Hf-UiO-67-FA-Pt after feeding water show an initiation period of approximately 5 h, where the selectivity to methanol increases, which will be discussed in §5 for all materials (electronic supplementary material, figure S15). A linker insertion procedure was performed on both Hf-UiO-67-FA-Pt and Zr-UiO-67-FA-Pt to investigate the effects of reducing the amount of missing linker defects in the materials and how it affects the catalytic stability. The evolution of methanol and CO yields is presented in figure 5, and the TOS performance during testing in the wet feed is shown in the electronic supplementary material, figure S13. Similarly to the other materials, methanol and CO yields are reduced during and after exposure to the wet feed. The previously sudden loss of methanol yield, which occurred after 10 and 12 h for Hf-UiO-67-FA-Pt and Zr-UiO-67-FA-Pt, respectively, now occurs after 4 h for both materials. A blank experiment was performed, using Hf-UiO-67-BA-Pt, to verify that reduced catalytic stability was caused by the reduction of missing linker defects and not the procedure of linker insertion itself. This sample is denoted Hf-UiO-67-BA-Pt-blank (refer to §2), and the results are presented in the electronic supplementary material, figure S14 and are within experimental uncertainty, the same as for Hf-UiO-67-BA-Pt.

To further investigate the water-induced deactivation, one of the catalysts, Hf-UiO-67-BA-Pt, was exposed to pretreatment under an inert atmosphere at 350°C after testing in the dry feed, wet feed and dry feed repeated. The catalyst was then tested at 210°C under the same conditions to compare the conversion of CO_2_ (electronic supplementary material, figure S20). A slight decrease in activity is observed when comparing the activity in the dry and wet feeds, from 1.9% to 1.7%. Exposure to the wet feed further decreased the activity to 1.3%, while the pretreatment at 350°C enhanced the CO_2_ conversion (1.6%).

Characterization of the samples after testing with water showed that for Zr-UiO-67-FA-Pt, Zr-UiO-67-BA-Pt and Hf-UiO-67-BA-Pt, the characteristic reflections of UiO-67 are still present and the materials are crystalline. Conversely, the Hf-UiO-67-FA-Pt material is less stable and is amorphous after catalytic testing (electronic supplementary material, figure S21). The nitrogen adsorption isotherms of the tested materials are shown in the electronic supplementary material, figure S22, and the surface areas, determined by BET, before and after catalytic testing with water in the reaction feed are reported in the electronic supplementary material, table S6. Similarly, as reported for the PXRD results, the framework of Hf-UiO-67-FA-Pt has collapsed, and only 10% of the surface area is retained after catalytic testing with water. Zr-UiO-67-BA-Pt and Zr-UiO-67-FA-Pt show a reduction in surface area, where approximately 70% and 80% of the surface area is retained after catalytic testing. Conversely, after testing, the Hf-UiO-67-BA-Pt sample retains 100% of the specific surface area. SEM images obtained after catalytic testing in the dry feed reveal intergrown octahedral crystallites (electronic supplementary material, figure S23). Furthermore, after testing in the wet feed, SEM images of the materials reveal that the crystallites are more deformed and intergrown. Pt NPs were solely detected for Zr-UiO-67-BA-Pt after exposure to the wet feed, with sizes ranging from approximately 6 to 9 nm (electronic supplementary material, figure S24).

### (d) DFT calculations

**Figure 6.**
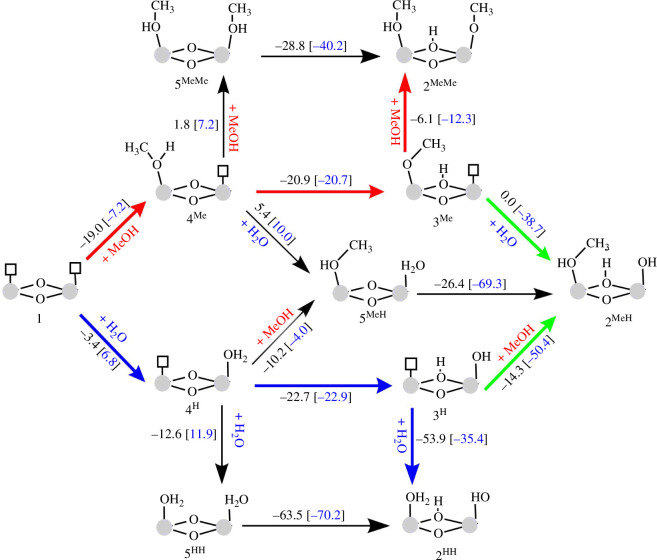
Free energy pathway (*T* = 170°C, *p* = 1 bar) representing the progression of competitive methanol and water adsorption or proton transfers on a defective Hf/Zr node. Reaction-free energies are given between each reaction step, with free energies for the Hf node shown in black and Zr node shown in blue and parentheses from our previous work [17]. All energies are in kJ mol^−1^.

The test results reported above suggest that the zirconium UiO-67 materials may be more stable than their hafnium equivalents. Furthermore, deactivation was only observed when water was co-fed, strongly indicating that water plays a role in the deactivation of the materials. To investigate this effect, we calculated the free energy for the adsorption of water and methanol on the hydrated Zr/Hf node with one missing linker labelled as (1) using the same DFT method and models previously reported with zirconium nodes [17]. Initially, the Hf node with one missing linker labelled as (1) in figure 6 contains two vacant sites and two *μ*_3_*–*O bridges. Coordination of water and/or methanol on structure (1) followed by proton transfer reactions generates 11 intermediates, as shown in figure 6. These intermediates are labelled using numbers to indicate species with different substituents at Zr/Hf sites and superscripts of H and/or Me to indicate whether water and/or methanol are present at the vacant sites, respectively.

From these energies, we can conclude that thermodynamics mainly dictates the preferred pathway. In the presence of MeOH, the formation of the most stable intermediate 2^MeMe^, where methanol is adsorbed on one of the M sites (M-CH_3_OH, M = Hf or Zr) and a methoxy is adsorbed on the other M site (M-OCH_3_) with a proton on one of the *μ*^3^–O sites (*μ*^3^–OH), is exergonic by 46.0 and 40.2 kJ mol for Hf and Zr nodes, respectively. An analogous process with water (1+H_2_O+H_2_O→2^HH^) is exergonic by 79.5 kJ mol^−1^ for Hf nodes and 51.5 kJ mol^−1^ for Zr nodes, which is 33.5 and 11.3 kJ mol^−1^ lower than with methanol for Hf and Zr nodes, respectively. However, in the presence of both methanol and water at equal concentration, intermediate 2^MeH^ is most favourable for Zr nodes with a Δ*G* of −66.5 kJ mol^−1^, while intermediate 2^HH^ is most favourable in the case of Hf nodes with a Δ*G* of −79.5 kJ mol^−1^.

We previously calculated energy barriers for the reactions in figure 6 using Zr nodes [17] and found that the barriers obtained were below 25 kJ mol^−1^ for proton transfer reactions and 0 kJ mol^−1^ for methanol and water adsorption on vacant sites. Using these energies, we built a MKM that agreed with 2^MeH^ as the preferred product. We built a similar MKM for Hf nodes, considering barrierless processes for methanol and water adsorption on vacant sites. The barriers for the proton transfer reactions at Hf nodes were evaluated using the Brønsted–Evans–Polanyi relationship [40,41] with a slope of 0.5 for activation energy versus potential energy, similar to what has been shown for proton–electron transfer reactions [42,43]. The MKM confirmed 2^HH^ as the preferred product at equal methanol and water concentrations with the Hf nodes.

## 5. Discussion

In this article, two different node metals, Hf and Zr, and two modulators, formic acid and benzoic acid, have been used to synthesize four UiO-67 materials with missing linker defects ranging from 1.0 to 2.0 per node. Both formate samples contained fewer missing linker defects than the benzoate equivalents. Notably, ^1^H NMR spectra obtained after catalytic testing show that the capping agents are entirely removed (electronic supplementary material, table S5). Therefore, the modulator’s impact on the activity is minimal, yet its choice affects the number of open Zr or Hf node sites. In the absence of water, the materials tested were stable at 170–240°C and 30 bars. However, in the wet feed, the materials showed widely different degradation, ranging from 100% retained surface area to 10%. No direct correlation between the framework stability and the node metal or missing linker defects was found.

When considering the formation rate of the different products, the hafnium materials show a higher rate towards methanol, with 10 and 14 mol_methanol_ mol_Pt_^−1^ h^−1^ for Hf-UiO-67-FA-Pt and Hf-UiO-67-BA-Pt compared with 6.2 and 5.8 for Zr-UiO-67-FA-Pt and Zr-UiO-67-BA-Pt at 240°C, respectively. However, the experimentally determined apparent activation energies for the material are the same for both hafnium and zirconium (44–52 kJ mol^−1^), indicating that the higher formation rate of hafnium is due to more active sites, potentially due to smaller Pt NPs or better contact between the hafnium nodes and the Pt NPs. Therefore, the assumption that the hafnium materials should facilitate the formation of methanol due to higher Lewis acid strength (refer to §1) is not supported by the experimental results presented in this work. The comparison of the product distribution for the hafnium and zirconium samples, shown in figure 1, indicates a similar product distribution for the catalysts. Therefore, we conclude that changing the node metal from Zr to Hf had no significant effect on the selectivity of the various products.

Experimentally, it was previously observed for Zr-UiO-67-Pt that the selectivity to methanol was reduced when the number of missing linker defects was reduced [17]. The same trend is observed for the hafnium materials tested in this study, where Hf-UiO-67-BA-Pt (1.4 missing linkers per node) show a slightly larger selectivity to methanol compared with Hf-UiO-67-FA-Pt at 170, 190 and 210°C (1.0 missing linkers per node). The same comparison could not be made for the zirconium samples due to a similar number of missing linker defects. Furthermore, it is important to note that the relationship between linker defects and methanol selectivity does not need to be linear, as DFT results suggest that the growth of Pt NPs facilitates linker detachment from the Zr nodes in the proximity, resulting in isolated zirconium nodes decorating the surface of the Pt NPs [16].

**Figure 7.**
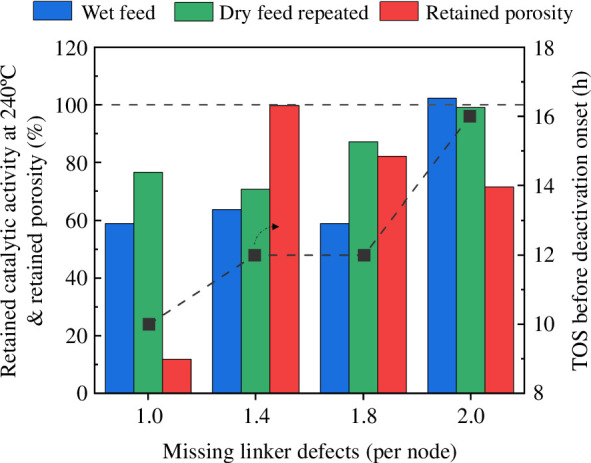
*Left axis*: retained catalytic activity at 240°C after exposure to the wet feed (blue column), subsequent exposure to the dry feed repeated (green column) and retained porosity after exposure to the wet feed (red column). *Right axis*: catalytic stability (black line + symbol) for Hf-UiO-67-FA-Pt (1.0 missing linker defect per node), Hf-UiO-67-BA-Pt (1.4 missing linker defects per node), Zr-UiO-67-FA-Pt (1.8 missing linker defects per node) and Zr-UiO-67-BA-Pt (2.0 missing linker defects per node).

To better understand the reason for catalytic deactivation on the wet feed, the retained porosity and catalytic activity at 240°C after exposure to the wet feed and subsequently the retained activity at 240°C when tested under the dry feed for a second time are represented in figure 7. We found no direct correlation between the TOS prior to the water-induced deactivation and the framework stability, as indicated by the retained surface area. Interestingly, Hf-UiO-67-FA-Pt, which was the least stable material tested (10% retained porosity), does not exhibit a significant reduction in methanol yield when comparing the test results in the dry feed before and after testing with water, indicating that the catalytic activity does not significantly depend on the porosity of the framework. This result is in line with our finding that pyrolysed samples of UiO-67-Pt, with a 10-fold reduction in uptake of nitrogen, showcase similar activity for CO_2_ hydrogenation to the pristine Zr-UiO-67-Pt material [18]. Similarly, a recent review article on CO_2_ hydrogenation over Pt-, Cu- and CuZn-containing Zr MOFs showed no correlation between the catalyst porosity and CO_2_ conversion [44]. However, we did find a positive correlation between the catalytic stability and the number of missing linker defects. The faster water-induced deactivation of the linker-inserted materials further supports this correlation. These results suggest that the active sites are more sensitive to water at a lower number of linker defects. Indeed, we found that water adsorption energies change significantly when the node has more than one linker defect [17].

Turning next to the influence of deactivation on product selectivity, a closer examination of the yields presented in figures 3 and 4 and the electronic supplementary material, figure S10, shows that although the yield of CO is lowered during and after exposure to the wet feed, it follows the expected Arrhenius-type behaviour with increasing yield under all conditions (figure 4). Conversely, the yields of methanol and methane show a different behaviour, i.e. both show a sudden yield decrease at either 210 or 240°C when water-induced deactivation occurs (figure 3 and electronic supplementary material, figure S10). The sudden reduction of methanol and methane yields suggests that the water-induced deactivation primarily affects the formation rate of methanol and methane. As discussed in §1, methane and methanol formation rates depend on the open zirconium sites near the Pt NPs. By contrast, the RDS of CO formation is the desorption of CO from the Pt NPs. These results indicate that there are two changes in the material, both an irreversible change affecting the overall activity of the material and a reversible change. The reversible change primarily affects the formation of methanol and methane and is reversible when exposed to a dry feed and higher temperatures (350°C). A plausible explanation for the irreversible deactivation of the materials in the presence of water could be the sintering of either the Pt NPs or the isolated zirconium nodes on the surface of the Pt NPs facilitated by the presence of water vapour.

Turning finally our attention to the initiation period observed when switching from the wet to the dry feed, a progressively increasing methanol yield is observed for all materials, albeit over varying durations. Previous experimental results show that if water is introduced into the feed over Zr-UiO-67-Pt during CO_2_ hydrogenation, a spike in methanol formation is observed before reaching a steady state. In the same work, Zr-UiO-67-Pt was saturated with water before switching to a CO_2_ + H_2_ reaction feed. The results showed an initiation period of 2 h before a steady state of methanol formation occurred. In the electronic supplementary material, figure S15, the TOS performance after water has been co-fed is shown for Zr-UiO-67-FA-Pt, Zr-UiO-67-BA-Pt, Hf-UiO-67-FA-Pt and Hf-UiO-67-BA-Pt. For all materials, a steady state is reached within the first 4 h on stream before the temperature is raised to 190°C except for Hf-UiO-67-FA-Pt, which required approximately 5 h. The initiation period is the shortest for the two zirconium materials, with roughly 20 min for Zr-UiO-67-BA-Pt and 1 h for Zr-UiO-67-FA-Pt. These findings are supported by the DFT calculations, which showed that water is more strongly bound to the hafnium nodes. For the Hf-UiO-67-BA-Pt catalyst, a steady state was not achieved before 1.5 h, and for Hf-UiO-67-FA-Pt, it took almost 5 h. Interestingly, the time to reach a steady state also seems to increase when the number of missing linker defects decreases. Perhaps more interesting, the materials that showed the shortest initiation periods were also the materials that were stable for longer during the catalytic test with water, indicating that the adsorption strength of water is crucial for the catalytic stability in the wet feed.

## 6. Conclusion

We have investigated hafnium and zirconium UiO-67 functionalized with Pt NPs for CO_2_ hydrogenation and applied PXRD, nitrogen adsorption, TGA and ^1^H NMR to evaluate the framework stability of these catalysts with 2 vol% water in the reaction feed. The prepared Hf-UiO-67-Pt catalysts displayed a superior formation rate of methanol per gram of Pt compared with the previously thoroughly studied Zr-UiO-67-Pt materials. However, the experimentally determined activation energy for methanol was similar for the hafnium and Zr materials, indicating that the increase in the formation rate of methanol for the hafnium sample is due to an increased number of active sites and not an increase in the TOF. Additionally, altering the metal in the node did not significantly impact the distribution of the products.

For all tested materials except Zr-UiO-67-BA-Pt, both irreversible and reversible water-induced deactivation was observed. The irreversible deactivation affected both the yield of CO and methanol. Conversely, the reversible change, which occurs after 4–12 h of testing in the wet feed, primarily affects the yield of methanol and methane. Based on previously published results, it is known that the formation rate of methanol and methane depends on the number of open zirconium sites. Conversely, the RDS of CO formation is the desorption of CO on the Pt NPs. Together, these findings indicate that water reversibly interacts with the open zirconium sites, while irreversible deactivation might involve the node as well as Pt NPs. No direct correlation was found between the stability of the frameworks and the irreversible or reversible water-induced deactivation. However, a positive correlation was found between the number of missing linker defects and the catalytic stability of the materials, although the precise reason behind this correlation remains unclear.

Experimental and computational results show that the heat of water adsorption is larger for the hafnium than the zirconium nodes, which could be a possible reason for the reduced stability of the hafnium catalysts in water.

## Data Availability

Data underlying the results reported in the manuscript are provided in the electronic supplementary material [45].
